# Invalidity of SUV Measurements of Lesions in Close Proximity to Hot Sources due to “Shine-Through” Effect on FDG PET-CT Interpretation

**DOI:** 10.1155/2012/867218

**Published:** 2012-10-14

**Authors:** Yiyan Liu

**Affiliations:** Nuclear Medicine Service, Department of Radiology, New Jersey Medical School, 150 Bergen Street, H-141, Newark, NJ 07103, USA

## Abstract

It is well known that many technical and physiologic factors can affect the reliability of the standardized uptake value (SUV) on FDG PET-CT. Another potential problem of which we may be aware but has not been previously discussed is significant SUV overestimation of lesions in the direct neighborhood of large hot sources, namely, areas with high FDG uptake or activity such as a tumor, myocardium, urinary bladder, kidney, or gastrointestinal tract. The magnitude of SUV overestimation of the lesions directly neighboring the large hot sources is varied among the different cases, and it is possibly secondary to “shine-through” effect of the hot sources, which would warrant further systematic investigation such as phantom simulation experiment. If the lesion is in the close territory of the hot source, measured SUV is often overestimated and invalid. Visual interpretation should be used for evaluation of FDG avidity of the lesion.

## 1. Introduction

Positron emission tomography (PET)/computed tomography (CT) with flurorine-18 fluorodeoxyglucose (FDG) has become an established imaging tool in oncology and is now of growing interest in inflammatory/infectious, cardiac, and neurological diseases. FDG PET data are normally assessed visually or by using simple indices such as the standardized uptake value (SUV) for quantification. An SUV is a semiquantitative number that normalizes lesion uptake to injected dose per unit of body mass. More generally, SUV may be normalized to other measure of body habitus such as lean body mass or body surface area. While many alternatives have been proposed, the SUV is generally evaluated at its maximum value as SUV_max⁡_. In practice, lesion SUV is determined by placing the region of interest (ROI) over the lesion and using computer program to automatically calculate the value [1]. SUVs are widely used to measure metabolic activity in lesions. Today, SUV measurements are increasingly being recognized as providing an objective, more accurate, and less observer-dependent measure for prognosis and response monitoring purpose than visual inspection alone [2, 3].

However, many technical and physiologic factors can affect the reliability of SUV, which include the blood glucose level, the time interval between FDG injection and image acquisition, patient's body composition and habitus, reconstruction technique, selection of region of interest, size of lesion, and the use of contrast agents during CT-attenuation correction. All these are well known to nuclear radiologists and have been discussed extensively in the literature [4–9]. Except for these, another potential problem of which we may be aware but has not been previously discussed is significant SUV overestimation of lesions in the direct neighborhood of large hot sources, namely, areas with high FDG uptake or activity such as a tumor, myocardium, urinary bladder, kidney, or gastrointestinal tract. If the lesion is close enough to a hot source, the SUV measurement may be invalid and misleading, and visual interpretation is much more reliable than the SUV number.

## 2. Materials and Methods

All image examples were from the PET-CT database in the Advanced Imaging Center, The University of Medicine and Dentistry of New Jersey. The review of PET-CT database was approved from the Institutional Review Board. Combined PET-CT was performed using a PET-CT scanner (Discovery LS, GE Healthcare) and standard techniques. The patients fasted for a minimum 6 hours before PET acquisition. After confirmation of a blood glucose level <200 mg/dL, 555 MBq (15 mCi) of sterile FDG was administered intravenously followed by a radiotracer uptake phase of approximately 60 minutes. Positron emission data sets were acquired from the base of the skull to the mid thigh, for 5 minutes at each bed position. PET images were reconstructed using the OSEM (ordered subset expectation maximization) algorithm. Low-dose CT was acquired and used for attenuation correction and was fused onto the PET images for anatomic correlation. To measure SUV, a 3D ROI is positioned centrally within a lesion or target using the interactive workstation. SUV_max⁡_ is recorded since it represents the highest voxel value and is independent of ROI definition [10].

PET-CT image examples were from 3 patients. Patient 1 was a 68-year-old man and had prostatectomy for prostate cancer 5 years ago. The recent serum prostate-specific antigen was negative. A routine chest radiography and subsequent chest CT revealed a 2.0 cm nodule in the lingula of the left lung. The PET-CT was for characterization of the pulmonary nodule.

Patient 2 was a 62-year-old woman and had history of cervical cancer. She underwent chemoradiation two years ago. She was asymptomatic, and the PET-CT was for surveillance.

Patient 3 was a 48-year-old man who was newly diagnosed with follicular carcinoma of the thyroid. The PET-CT was for initial staging.

## 3. Results

**Figure 1:**
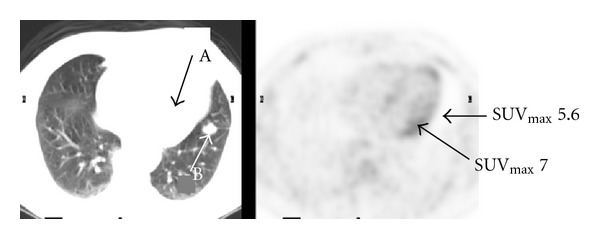
Transaxial FDG PET-CT images of the chest show physiologic myocardial uptake with SUV_max⁡_ 7.0 (arrow A) and no visible uptake in a 2.0 cm lingular nodule of the left lung (arrow B). However, measured SUV_max⁡_ of the nodule is 5.6.

**Figure 2:**
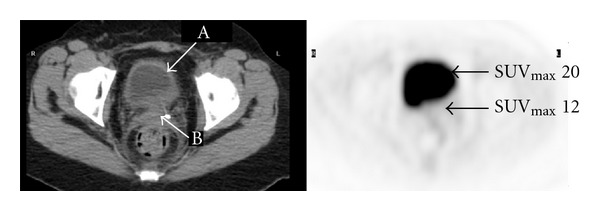
Transaxial FDG PET-CT images of the pelvis show no abnormal uptake in the endocervix of a woman status after chemoradiation for cervical cancer. However, measured SUV_max⁡_ is 12 in the endocervix (arrow B). Urine SUV_max⁡_ in the bladder is 20 (arrow A).

**Figure 3:**
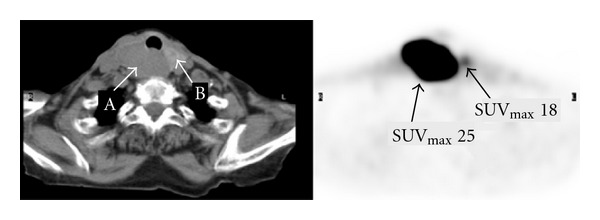
Transaxial FDG PET-CT images of the neck show a large lesion with SUV_max⁡_ 25 in the right lobe of the thyroid (arrow A). There is a 1.5 cm nodule with mild to moderate uptake in the left lobe, but unexpectedly measured SUV_max⁡_ is 18 (arrow B).

Figures 1–3 represent selected axial images of the PET-CT from 3 case examples, which all demonstrate overestimations of SUV_max⁡_ of the lesions directly neighboring large hot sources. In Figure 1, SUV_max⁡_ of the myocardium is 7.0, and measured SUV_max⁡_ of a 2.0 cm lingular nodule is 5.6 even though there is no visible uptake. Repeating FDG PET-CT six months later in the same patient shows the unchanged, non-FDG avid lingular nodule, but measured SUV_max⁡_ is 2.2 due to less intense cardiac uptake (SUV_max⁡_ 4.0) compared to the first scan (the images not shown). In Figure 2, there is no abnormal uptake in the endocervix in the patient with history of cervical cancer and after chemoradiation, but measured cervical SUV_max⁡_ is 12. The bladder urine SUV_max⁡_ is 20. The subsequent Pap smear was negative for recurrence of cervical cancer. In Figure 3, there is a large, highly FDG avid right-sided thyroid lesion with SUV_max⁡_ 25. A 1.5 cm nodule in the left lobe of the thyroid demonstrates much less uptake than the right, but measured SUV_max⁡_ is 18. In all of the three examples above, the SUVs of the lesions (lung nodule, endocervix, and left thyroid nodule) were unexpectedly overestimated due to their locations in close proximity to the large sources of high radioactivity in the myocardium, urinary bladder and right thyroid mass.

**Figure 4:**
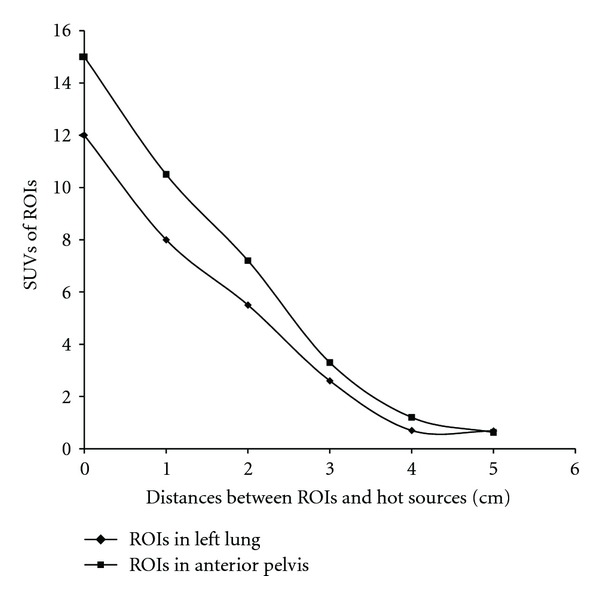
SUV changes with the distances between the ROIs and hot sources in two cases. The lower curve represents the SUV_max⁡_ of the ROIs in the left lung at the different distance from hot myocardium. The upper curve represents the SUV_max⁡_ of the ROIs in the anterior pelvis at the different distance from the urinary bladder.

Figure 4 shows dynamic changes of series SUVs when the distances between the ROIs and hot source increase. The two curves in the plot indicate that SUVs in the normal left lung and normal anterior pelvis decrease when the ROIs are away from hot sources myocardium and urinary bladder, respectively. In both examples, the SUVs decline to normal points when the ROIs are about 4–5 cm away from the hot sources. Therefore, SUVs in these two cases are overestimated and invalid if the ROIs are within 4–5 cm distance from hot sources.

## 4. Discussion

The uptake value is represented by pixel or voxel intensity value in the ROI of the image, which is then converted into the activity concentration. Overestimated SUV values of the lesion directly adjacent to the large hot source are likely secondary to “shine-through” effect. The “shine-through” effect is defined as the detection of radioactivity from a second source rather than the region of the interest or lesion [11]. In the three cases reported here, the second sources of radioactivity are from the cardiac, urinary bladder, and large lesion in the right lobe of the thyroid. Since the lesion of interest is close to the hot second source, the counts of radioactivity in the lesion may include the counts originating from the adjacent hot source such as a structure or another lesion rather than from the measured lesion only. Therefore, radioactivity is usually overestimated in a lesion in close proximity to a large hot source.

Although it is not previously discussed on SUV measurement in PET-CT interpretation, radioactivity “shine-through” effect had been recognized as a potential problem in lymph node mapping technique, in which a radiotracer is injected onto or around the primary tumor such as breast cancer and melanoma [11–16]. If the primary tumor is close to the lymph node basin, the radioactivity of the primary tumor may obscure identification of the sentinel lymph node. In other words, high radioactivity originating from the injection site may hinder the focal localization of the sentinel lymph node. It is difficult to determine if measured high radioactivity adjacent to the primary tumor/injection site is from sentinel node or secondary to “shine-through” effect. In sentinel lymph node mapping for breast cancer, the “shine-through” effect is the most prominent when the tumor is located in the outer upper quadrant [11], obviously due to the close proximity of the axillary sentinel node to the injection site. Although the concepts of the “shine-through” effects between the lymph node mapping and SUV measurement may not be completely the same, both contribute extraradioactivity from the second hot sources to measured or recorded radioactivity of the ROIs. In addition, “shine-through” artifact has been also reported as an interpretation pitfall of two different imagings of the same organ or region in nuclear medicine studies such as dual-tracer parathyroid scintigraphy [17].

It seems that the magnitude of “shine-through” effect is dependent on the radioactivity of the hot source, and distance from the hot source. The larger the hot source and the greater the radioactivity, the more extended the “shine-through” effect in its neighborhood territory. The closer the ROI to the hot source, the more prominent “shine-through” effect and the greater overestimated SUV. Figure 4 shows that SUVs in the normal left lung and normal anterior pelvis decrease when the ROIs are away from hot sources myocardium and urinary bladder, respectively. The SUVs decline to normal points when the ROIs are about 4–5 cm away from the hot sources in both case examples. Therefore, SUVs in these two cases are all overestimated and invalid if the ROIs are within 4–5 cm from the hot source. But obviously, the magnitude of the “shine-through” effect is case-based and varied with different hot sources.

The “shine-through” effect is not an imaging noise. It is not related to the size of the lesion and not partial volume artifact. It is independent of reconstruction techniques, either simple filtered-backprojection or iteration reconstruction algorithms.

## 5. Conclusion

Quantitative SUV measurement may be invalid due to the proximity of intense background sources to a lesion or region of interest, which is not a commonly discussed artifact in clinical interpretation but undoubtedly a potentially important one. If the lesion is close to a large hot source such as tumor, myocardium, or urinary bladder, measured SUV is often overestimated. Visual interpretation should be used for evaluation of FDG avidity of the lesion. The magnitude of SUV overestimation of the lesions directly neighboring the large hot sources is varied among the different cases, and it is possibly secondary to “shine-through” effect of the hot sources, which would warrant further systematic investigation such as phantom simulation experiment.
